# Development of a Highly Sensitive ELISA for Detecting Antibodies Against a Novel Variant Avian Reovirus Based on Dual σC and σB Antigens

**DOI:** 10.3390/ani16081273

**Published:** 2026-04-21

**Authors:** Fuxi Zhao, Wanyi Zhou, Yilin Yuan, Qiuyun Peng, Weibin Wang, Weisheng Cao

**Affiliations:** 1 College of Veterinary Medicine, South China Agricultural University, Guangzhou 510642, China; zfx951023@163.com (F.Z.); 18207631723@163.com (W.Z.); yuanyilin2022@163.com (Y.Y.); 18128356251@163.com (Q.P.); 15975227784@163.com (W.W.); 2 Key Laboratory of Zoonosis Prevention and Control of Guangdong Province, Guangzhou 510642, China; 3 Key Laboratory of Zoonosis of Ministry of Agriculture and Rural Affairs, Guangzhou 510642, China; 4 Key Laboratory of Veterinary Vaccine Innovation of the Ministry of Agriculture and Rural Affairs, Guangzhou 510642, China; 5 National and Regional Joint Engineering Laboratory for Medicament of Zoonosis Prevention and Control, Guangzhou 510642, China

**Keywords:** avian reovirus, dual-antigen indirect ELISA, antibody detection, σC protein, σB protein

## Abstract

This study developed a dual-antigen enzyme-linked immunosorbent assay (ELISA) based on σB protein and genotype 5-specific σC protein of avian reovirus (ARV). First, σB and σC proteins were expressed and purified using recombinant technology. Through optimization of coating conditions, the optimal antigen combination was determined to be a mixture of the two proteins at a 1:3 molecular ratio (total concentration: 0.8 μg/mL). Key parameters of the indirect ELISA were optimized via checkerboard titration. Validation confirmed that the dual-antigen ELISA exhibited a sensitivity of 1:3200 against genotype 5 ARV-positive sera, with no cross-reactivity and a coefficient of variation of 2.9–8.6%, demonstrating excellent reproducibility. In application testing, the method specifically detected serum antibodies against genotype 5 ARV variant strains, achieving a 100% positive detection rate in experimental chickens within the first week post-challenge and effectively monitoring dynamic antibody changes in infected flocks. Furthermore, the detection rate for genotype 5-positive serum samples (100%) was significantly higher than that of a commercial kit (75%). This dual-antigen indirect ELISA overcomes the sensitivity limitations associated with conventional genotype 5 ARV detection methods and provides a reliable tool for epidemiological surveillance and infection monitoring.

## Simple Summary

Avian viral arthritis, an economically significant disease caused by avian reovirus (ARV), poses a substantial threat to poultry health. The continuous evolution of ARV and the concurrent circulation of multiple genotypes have challenged the efficacy of current diagnostic approaches. Concurrently, the detection rate of genotype 5 ARV in Chinese chicken flocks has steadily increased, highlighting an urgent need to establish a rapid and efficient detection method for early diagnosis and timely control of ARV infections. To address this need, a novel dual-antigen indirect enzyme-linked immunosorbent assay (ELISA) was developed based on the ARV σB protein and the genotype 5-specific σC protein. The established assay demonstrated high specificity, sensitivity, and reproducibility, significantly improving the serological detection of genotype 5 ARV infections and enabling effective discrimination between antibodies induced by vaccine strains and those induced by field virus infections. The establishment of this detection system provides reliable technical support for monitoring ARV infection dynamics and conducting epidemiological investigations.

## 1. Introduction

Avian reovirus (ARV), a significant pathogen affecting global poultry production, poses a serious threat to avian health. The virus causes various wasting diseases [1,2], with viral arthritis/tenosynovitis representing the most clinically significant manifestation [3,4]. Phylogenetic analysis based on the σC protein-encoding gene indicates that ARV can be classified into at least seven distinct genotypes [5,6]. Notably, the infection range of genotype 5 ARV has continued to expand in recent years [7], and it has rapidly become a highly prevalent strain in China’s major poultry-producing regions [8,9,10]. The persistent circulation of this genotype underscores the urgent need for precise epidemiological surveillance and targeted control strategies.

Currently, there is no effective clinical treatment for ARV infection, making vaccination the primary preventive strategy. All registered vaccines are derived from a limited number of genotype lineage I strains. Multiple studies have demonstrated that novel or genetically distinct ARV variants have emerged that fail to induce sufficient neutralizing antibody titers in birds immunized with existing vaccine strains, thereby compromising protection against infection [11,12,13]. Furthermore, even vaccinated animals exhibit insufficient protection against field strains [14]. Since immunization with an isolate from one serotype (or genetic lineage) protects animals only against viruses from the same group, assessing the genetic variability of ARV is essential for developing scientifically sound and effective vaccination programs [15,16].

ARV belongs to the genus *Orthoreovirus* within the family Reoviridae [17]. Its genome comprises ten double-stranded RNA segments encapsulated within a double-layered capsid. These segments are categorized into three classes based on electrophoretic mobility: L (L1–L3), M (M1–M3), and S (S1–S4) [18,19]. The genome encodes 12 principal proteins, including four non-structural proteins (μNS, p10, p17, and σNS) and eight structural proteins (λA, λB, λC, μA, μB, σA, σB, and σC) [20,21,22]. The σB and σC proteins, encoded by the S3 and S1 gene segments, respectively, serve as key targets for the host humoral immune response. Functioning as the viral attachment protein, σC determines tissue tropism by binding to specific cell surface receptors [23]. As the most variable structural protein of ARV [24,25,26], σC harbors genotype-specific neutralizing epitopes that exhibit significant genetic diversity, forming the molecular basis for genotyping [27]. Antibodies against σC demonstrate strict genotype specificity with minimal cross-reactivity between phylogenetically distant strains, making it an ideal target for genotype-specific serodiagnosis. In contrast, σB, a core protein that mediates viral membrane penetration and fusion, is highly conserved across genotypes [28,29]. Antibodies targeting σB recognize broadly shared epitopes, establishing σB as a preferred antigen for pan-genotypic antibody detection.

Although clinical signs and postmortem lesions may provide preliminary diagnostic clues for viral arthritis, laboratory confirmation remains essential [30]. Currently, commercial tests based on viral lysates primarily detect non-neutralizing antibodies and cannot accurately reflect the actual immune status of poultry flocks [31,32]. As the gold standard for serological testing, enzyme-linked immunosorbent assay (ELISA) offers high sensitivity and specificity, significantly improving the efficiency of large-scale epidemiological screening. Detection methods employing highly immunogenic recombinant σB and σC proteins have demonstrated greater specificity than those using viral lysates and show good correlation with seroneutralization test results [24,33,34].

Given the high genetic variability of the ARV genome and the continuous emergence of new strains, the development of accurate and rapid diagnostic techniques is critical for the early diagnosis, outbreak control, and epidemiological investigation of ARV in poultry farms. Based on the distinct immunogenic properties of the σB and σC proteins, this study established a dual-antigen indirect ELISA method by systematically evaluating detection performance under different molar ratios (σB:σC). This method employs two antigens to synergistically detect target antibodies and, compared with existing methods, demonstrates enhanced sensitivity and specificity for genotype 5 variants. This technique is expected to provide essential support for ARV surveillance and to offer a scientific basis for the development of targeted prevention and control strategies.

## 2. Materials and Methods

### 2.1. Ethical Statement

This study strictly adhered to the Guidelines for the Welfare and Ethical Review of Laboratory Animals issued by the Ministry of Science and Technology of the People’s Republic of China. All animal experiments were reviewed and approved by the Laboratory Animal Management and Use Committee of South China Agricultural University. All procedures involving live viruses and animals were conducted in an Animal Biosafety Level 2 (ABSL-2) laboratory.

### 2.2. Experimental Materials

The *E. coli* BL21(DE3) cells, the pET-28a (+) vector, and mouse anti-ARV σB/σC polyclonal antibodies were available in-house. Two-week-old specific pathogen-free (SPF) chickens were supplied by Guangdong Xinxing Dahuanong Egg Industry, Yunfu, China. The representative ARV genotype 5 strain LY383 (A/chicken/Shandong/LY383/2017) was kindly provided by Prof. Youxiang Diao (Shandong Agricultural University). The laboratory repository provided 40 PCR-positive samples and the ARV genotype 5 strain RG5. Additionally, 40 clinical serum samples from chickens vaccinated with the S1133 live vaccine were collected from a poultry farm in Guangdong, China, for this study.

### 2.3. Plasmid Construction, and σC and σB Protein Expression and Purification

Based on the nucleotide sequence of the LY383 strain (GenBank accession number: AWV55509), the σC and σB gene sequences were synthesized by Sangon Biotech (Shanghai, China) and subsequently cloned into the pMD19T vector (Takara, Kyoto, Japan). Gene-specific primers were designed to amplify the σC gene using the pMD19T-σC plasmid as the template: forward primer pETσC-F (5′-GCTCGAGGGTATCAATGCCGGTGCGC-3′) and reverse primer pETσC-R (5′-GGCTAGCATGGACGGGCTGACCCAGC-3′), with underlined sequences indicating *Xho I* and *Nhe I* restriction sites. Similarly, primers for σB gene amplification (template: pMD19T-σB plasmid) were forward primer pETσB-F (5′-GCTCGAGCCATCCGCATTTCACCACG-3′) and reverse primer pETσB-R (5′-GGCTAGCATGGAGGTGAGAGTGCCC-3′), containing *Xho I* and *Nhe I* restriction sites (underlined). Following sequence verification, the σC and σB genes were individually ligated into the pET-28a (+) expression vector. The recombinant plasmid was transformed into *Escherichia coli* DH5α-competent cells (Tiangen Biotech, Beijing, China), followed by incubation with shaking at 37 °C and 220 rpm for 12–14 h in Luria–Bertani medium containing kanamycin (50 μg/mL). After optimizing the induction conditions, when the optical density (OD600) of the bacterial culture reached 0.6–0.8, 1.0 mM isopropyl β-d-1-thiogalactopyranoside (IPTG) was added to induce protein expression [35], and incubation was continued at 37 °C for 5 h. Subsequently, bacterial cells were collected by centrifugation at 12,000× *g* for 20 min at 4 °C. The bacterial pellet was resuspended in denaturing lysis buffer (1/10 the volume of the culture medium) and subjected to ultrasonic disruption. Recombinant σC and σB proteins were purified under denaturing conditions using nickel-affinity chromatography according to the manufacturer’s protocol (His-tag Protein Purification Kit, denaturant-resistant, Beyotime, Shanghai, China). The target proteins were refolded using a stepwise urea gradient (6 M, 4 M, 2 M) in phosphate-buffered saline (PBS) at 4 °C. Protein concentrations were quantified using a BCA assay kit (Beyotime, Shanghai, China).

### 2.4. SDS-PAGE and Western Blot Analysis

Following SDS-PAGE separation, the σC and σB proteins were electrophoretically transferred onto a polyvinylidene fluoride membrane (Merck, Darmstadt, Germany). The membrane was blocked using 5% skim milk in PBST (PBS containing 0.05% Tween-20) at 37 °C for 1 h [36]. Two parallel immunoblotting assays were conducted: one using a 1:200 dilution of reovirus-positive serum to verify that the recombinant protein could be specifically recognized by ARV-positive serum, thereby confirming its immunogenicity, and the other using a 1:500 dilution of a mouse anti-His monoclonal antibody (Beyotime, Shanghai, China) to detect the His tag on the recombinant protein. The membranes were then incubated with the corresponding secondary antibodies: IRDye^®^ 800CW Goat anti-Chicken IgG for the reovirus serum or IRDye^®^ 800CW Goat anti-Mouse IgG for the anti-His antibody (1:2000 dilution, both from LI-COR Biosciences, Lincoln, NE, USA). Protein signals were subsequently detected using an Azure Biosystems imager (Azure Biosystems, Inc., Dublin, CA, USA).

### 2.5. Establishment of the Indirect ELISA Method Based on σC and σB Dual-Antigens

To determine the optimal coating antigen, separate coating groups were established for σB antigen alone, σC antigen alone, and a mixture of σB and σC antigens, each evaluated at coating concentrations of 0.2, 0.4, 0.6, 0.8, 1, and 2 μg/mL. ARV-positive and ARV-negative sera diluted at a ratio of 1:200 were added to each well and incubated for 1 h. Subsequently, based on the optimal coating antigen, checkerboard titration was performed to further optimize the antigen coating concentration and serum dilution [37]. The σC and σB proteins were serially diluted in carbonate-bicarbonate buffer (0.05 M, pH 9.6) and coated onto 96-well plates at molar ratios ranging from 4:1 to 1:4, followed by overnight incubation at 4 °C. The coated plates were then incubated with gradient-diluted ARV reference positive and negative sera (1:50–1:1200) to determine the optimal coating conditions. For blocking optimization, 3%, 5%, and 8% solutions of skim milk or bovine serum albumin (BSA) were comparatively evaluated after incubation at 37 °C for 0.5–2 h. Systematic assessments included serum incubation time (0.5–2 h) and secondary antibody conditions (horseradish peroxidase [HRP]-conjugated goat anti-chicken IgY, 1:3000–1:10,000 dilution, incubation for 0.5–2.5 h; Abbkine, Wuhan, China). After the addition of TMB substrate (room temperature, 10–30 min; Solarbio, Beijing, China), the reactions were terminated with 100 μL of 2 M H_2_SO_4_. Optimal parameters were determined by selecting the conditions that yielded the maximum absorbance difference (OD_450nm_) between positive and negative controls, quantified as the highest positive-to-negative (P/N) ratio. All tests were performed in triplicate.

### 2.6. Determination of the Cutoff Value

To establish the cutoff value, the dual-antigen ELISA was conducted under optimized conditions using 30 ARV-negative chicken serum samples and 30 serum samples from chickens vaccinated with the S1133 live attenuated vaccine (MSD, Rahway, NJ, USA). The absorbance of each sample at 450 nm (OD_450_) was measured using an automated microplate reader, and the mean (X) and standard deviation (SD) were calculated. A sample was considered positive if its OD_450_ value exceeded X + 3SD; otherwise, it was classified as negative.

### 2.7. Determination of Diagnostic Sensitivity, Specificity, and Repeatability

The specificity of the established dual-antigen ELISA was validated using reference positive and negative sera against avian influenza virus (AIV), avian leukosis virus (ALV), Newcastle disease virus (NDV), infectious bursal disease virus (IBDV), and ARV. For sensitivity evaluation, positive serum was subjected to two-fold serial dilutions ranging from 1:200 to 1:6000. The highest dilution that yielded a positive result (i.e., an absorbance value above the cutoff) was defined as the limit of detection (LOD). To assess repeatability (intra-assay variability), six positive and six negative serum samples were analyzed in triplicate using the dual-antigen ELISA. The mean, standard deviation (SD), and coefficient of variation (CV = SD/mean × 100%) were calculated for each sample. To evaluate reproducibility (inter-assay variability), the same six serum samples were tested on three different 96-well plates coated with different antigen batches on separate occasions. The mean and SD of the OD_450_ values were determined, and the CV was calculated accordingly.

### 2.8. Applicability Testing of the ELISA Method

To evaluate the detection efficacy of the established ELISA method, 2-week-old SPF chickens were immunized with genotype 5 strains (LY383 and RG5) and the S1133 vaccine strain (10^5^ TCID_50_ per bird). Serum samples were collected weekly and analyzed using this ELISA, and the resulting data were subsequently evaluated.

### 2.9. Comparison of Dual-Antigen Indirect ELISA with Commercial Kits

A parallel comparison was conducted using both the dual-antigen ELISA and a commercial ARV antibody detection kit (Keaobo, Shanghai, China) on 40 genotype 5 ARV-positive serum samples and 30 confirmed ARV-negative serum samples. The positive and negative concordance rates between the two methods were subsequently calculated based on the results.

## 3. Results

### 3.1. Expression, Purification, and Immunogenicity Analysis of Recombinant σB and σC Proteins

**Figure 1:**
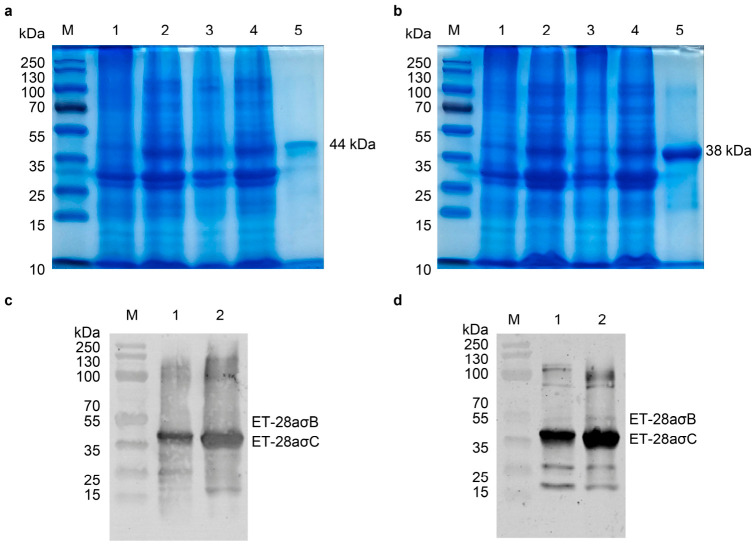
Solubility analysis and purification of ET28a-σB and ET28a-σC: (**a**,**b**) Solubility profiles of ET28a-σB (**a**) and ET28a-σC (**b**). M: protein marker; lane 1: uninduced sample; lane 2: induced sample; lane 3: supernatant after ultrasonication; lane 4: pellet after ultrasonication; lane 5: purified protein. (**c**) Western blot of ET28a-σB and ET28a-σC probed with ARV-positive serum as the primary antibody. M: protein marker; lane 1: ET28a-σB protein. (**d**) Western blot using a mouse anti-His monoclonal antibody as the primary antibody. M: protein marker; lane 1: ET28a-σB protein; lane 2: ET28a-σC protein.

The expression of pET-28aσB and pET-28aσC was optimized by transforming the respective plasmids into *E. coli* BL21(DE3) cells. Expression was induced with 1 mM IPTG at OD_600_ values of 0.584 (σB) and 0.569 (σC), followed by incubation for 5 h. After cell lysis and centrifugation, SDS-PAGE analysis revealed that the σB protein (~44 kDa) was present in both soluble and insoluble fractions, whereas σC (~38 kDa) was predominantly detected in the insoluble fraction. Both proteins corresponded to their predicted molecular weights (Figure 1b,c). The purified and refolded proteins were subsequently analyzed by Western blot. When probed with ARV-positive serum/goat anti-chicken IgY (Figure 1c) or mouse anti-His monoclonal antibody/goat anti-mouse IgG (Figure 1d), specific bands were observed at 38 kDa and 44 kDa, confirming specific antibody recognition and demonstrating favorable immunogenicity.

### 3.2. Establishment of an Indirect ELISA Method Based on σC and σB Dual-Antigen

The optimal coating concentrations for the ET28a-σC and ET28a-σB proteins, both individually and in combination, were determined using a checkerboard titration assay. The results indicated that a mixed-antigen concentration of 0.8 μg/mL (ET28a-σC and ET28a-σB) produced the greatest difference in optical density (OD) between positive (P) and negative (N) sera, achieving a P/N ratio of 12.333 (Table 1). Accordingly, the optimal coating concentration was established as 80 ng per well. Further optimization based on the molar ratio of the two proteins identified an optimal coating ratio of 1:3 for ET28a-σB to ET28a-σC (Table 2).

**Figure 2:**
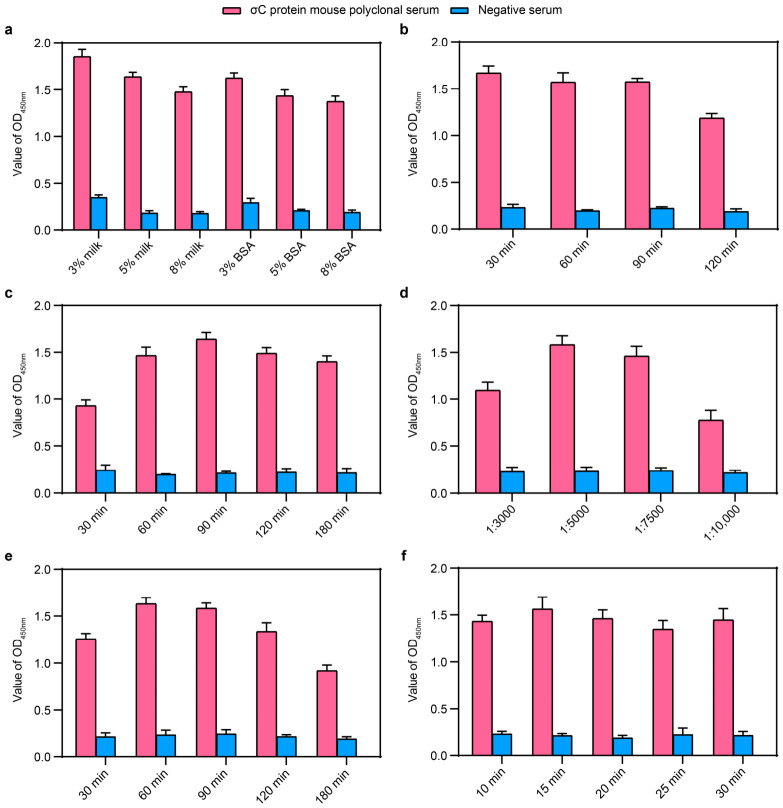
Optimization of dual-antigen indirect ELISA procedure: (**a**) Selection of optimal blocking solution; (**b**) Determination of optimal blocking duration; (**c**) Optimization of serum incubation time; (**d**) Determination of optimal secondary antibody dilution ratio; (**e**) Optimization of secondary antibody incubation time; (**f**) Determination of optimal chromogenic reaction time.

In this study, various reaction conditions were systematically optimized using a dual-antigen indirect ELISA. The optimal protocol was established as follows: coating with ET28-σB and ET28-σC antigens at a molar ratio of 1:3 in carbonate buffer, blocking with 5% skimmed milk at 37 °C for 60 min (Figure 2a,b), serum incubation at 37 °C for 90 min (Figure 2c), incubation with HRP-conjugated secondary antibody at a dilution of 1:5000 for 30 min (Figure 2d,e), and finally, color development at 37 °C for 20 min protected from light (Figure 2f).

### 3.3. Determination of the Critical Value

**Figure 3:**
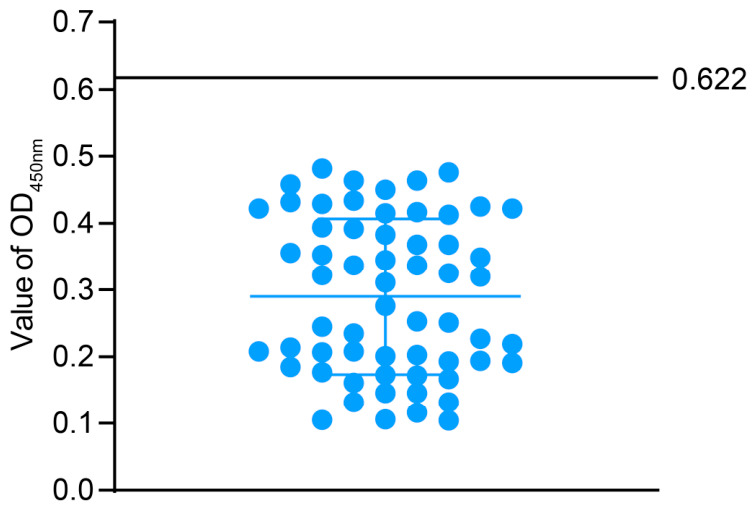
Determination of the cutoff value of the dual-antigen indirect ELISA (OD_450_ 0.622).

Using this method, 30 SPF chicken negative serum samples and 30 serum samples from chickens vaccinated with the ARV S1133 live vaccine were tested. Based on the OD_450nm_ values obtained, the mean value of the 60 samples was calculated as 0.286, with a standard deviation of 0.112. The final cutoff value was defined as the mean plus three times the standard deviation ($\overset{—}{X}$ + 3SD), corresponding to 0.622 (Figure 3). Accordingly, the criterion for positive/negative determination in this method is as follows: a sample with an OD_450nm_ value ≥ 0.622 is considered positive; otherwise, it is considered negative.

### 3.4. Assessment of Sensitivity, Specificity, and Repeatability in Dual-Antigen ELISA

**Figure 4:**
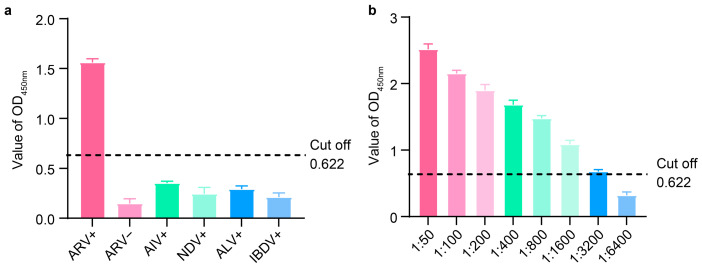
Sensitivity and specificity of the dual-antigen indirect ELISA. (**a**) Specificity test of the dual-antigen indirect ELISA. The assay detected no cross-reactions with sera containing antibodies against four other avian pathogens, including AIV, ALV, NDV, and IBDV. (**b**) Determination of sensitivity.

ELISA was performed on positive samples of AIV, NDV, ALV, IBDV, and ARV, as well as ARV-negative serum samples preserved in the laboratory. The results demonstrated that only the ARV-positive samples showed a positive reaction, whereas all other samples were negative (Figure 4a), indicating that the method exhibits good specificity. Furthermore, positive serum was subjected to gradient dilution, and serum samples at different dilution levels were analyzed by ELISA. The results showed that even at a dilution of 1:3200 (Figure 4b), the detection result remained positive, indicating high sensitivity.

To evaluate the repeatability of the dual-antigen ELISA method, intra-assay and inter-assay tests were performed using six positive serum samples. The inter-assay coefficients of variation (CV) ranged from 2.9% to 5.9%, whereas intra-assay CV values ranged from 5.6% to 8.6% (Table 3). These results indicate that the assay exhibits high repeatability.

### 3.5. Evaluating the Application of the ELISA Method

**Figure 5:**
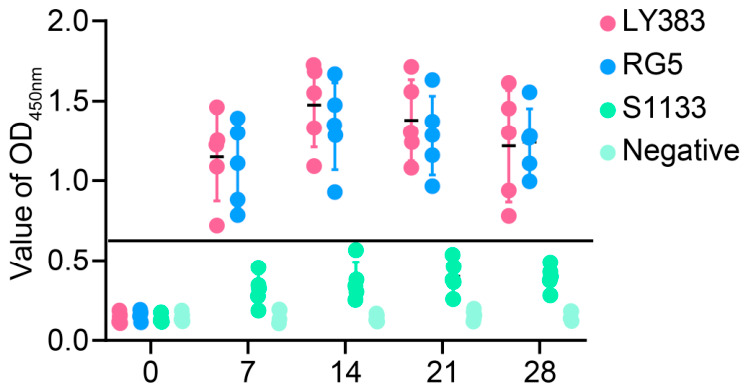
Results of serum antibody detection in ARV variant- and S1133-infected chickens.

Using the established ELISA method, positive serum samples from SPF chickens infected with ARV variant strains LY383 and RG5, as well as the vaccine strain S1133, were tested. Phylogenetic relationships of σC protein in genotype 5 ARV strains (including LY383 and RG5) are shown in Figure S1. The results demonstrated that this indirect ELISA method specifically detected serum antibodies against the genotype 5 ARV variant strains, achieving a 100% positive detection rate within the first week post-challenge (Figure 5). Additionally, it effectively reflected the dynamic changes in antibody levels in infected chickens. These findings indicate that the dual-antigen indirect ELISA method developed in this study possesses important practical value.

### 3.6. Detection of Clinical Samples

The established dual-antigen ELISA method was used to detect 40 positive serum samples and 30 negative serum samples provided by the laboratory. The results showed that this method exhibited higher sensitivity than the commercial kit (Table 4), with a sensitivity of 100% (40/40) and a specificity of 100% (30/30). In contrast, the commercial kit showed a sensitivity of 75.0% (30/40) and a specificity of 100% (30/30) on the same sample set. These results indicate that the ELISA method developed in this study is suitable for the effective detection of ARV in clinical serum samples.

## 4. Discussion

ARV is globally distributed and poses a serious threat to poultry health. Despite the widespread implementation of vaccination, vaccinated flocks often fail to develop sufficient neutralizing antibody titers to resist infection because of substantial antigenic differences between vaccine strains and circulating field strains, resulting in incomplete immune protection [6,38]. Commercial diagnostic methods for ARV infection commonly use viral lysates as coating antigens. Although these assays can detect total antibodies against the pathogen, most of these antibodies lack protective efficacy [39]. Moreover, due to the high variability of the σC protein, such methods often exhibit insufficient sensitivity and lack genotype-specific detection capability. Both the σB and σC proteins can induce the production of neutralizing antibodies, and monoclonal antibodies targeting these proteins effectively inhibit viral infection in cell culture [40]. Yang et al. [34] fused the σB and σC proteins of strain S1133 as coating antigens, establishing an indirect ELISA that achieved 100% concordance with the virus neutralization test, compared with 95.8% for traditional ELISA. To address the potential sensitivity limitations associated with the use of a single recombinant protein, this study developed a dual-antigen indirect ELISA based on σB and σC proteins. The design of this dual-antigen system is based on the differences in the biological functions of the σB and σC proteins, as well as their synergistic effects within the immunoassay system. The σC protein, as the most variable structural protein of ARV, contains genotype-specific neutralizing epitopes. In contrast, the σB protein is highly conserved across ARV genotypes and can induce broadly cross-reactive antibodies, serving an auxiliary diagnostic role. Consequently, previous studies have demonstrated that co-expression of σC and σB proteins produces a complementary effect, in which σB may enhance overall immunogenicity or confer broader protection [15,34,40]. By combining these two antigens, the established ELISA achieves a synergistic effect: σC ensures genotype-specific recognition of ARV, whereas σB enhances overall antibody capture efficiency, thereby overcoming the trade-off between specificity and sensitivity inherent in single-antigen detection methods. Optimization of the antigen molar ratio (σB:σC = 1:3) and coating concentration (0.8 μg/mL) further confirms the importance of rational antigen combination in improving assay performance.

Performance validation results demonstrated that the dual-antigen ELISA exhibits excellent sensitivity, with an LOD of 1:3200 for genotype 5 ARV-positive sera. This feature is particularly important for detecting low-titer antibodies during early infection stages or in flocks with incomplete immune protection. In clinical sample testing, the method achieved a 100% detection rate for positive sera, significantly outperforming the 75% detection rate of the commercial kit. This advantage is attributed to the capacity of recombinant σC and σB proteins to specifically recognize neutralizing antibodies and conserved epitopes of genotype 5 ARV, respectively, unlike commercial kits that primarily target non-neutralizing antibodies in viral lysates [41]. Furthermore, the assay showed no cross-reactivity with sera from other avian pathogens (e.g., AIV, NDV, ALV, IBDV), demonstrating high specificity and enabling accurate differentiation between ARV infection and other avian diseases. Additionally, the dual-antigen ELISA exhibited good reproducibility, with intra-assay CV ranging from 2.9% to 5.9% and inter-assay CV ranging from 5.6% to 8.6%, meeting the technical requirements for large-scale epidemiological surveys.

In SPF chickens infected with genotype 5 ARV strains (LY383 and RG5) and the S1133 vaccine strain, the method consistently detected dynamic changes in antibody levels, with antibody elevation detectable as early as 1 week post-infection, providing an effective tool for evaluating immune responses following vaccination or natural infection. Notably, the ability of this method to monitor antibody dynamics in vaccinated flocks addresses the urgent need for vaccine efficacy assessment, particularly in the context of the increasing prevalence of genotype 5 strains capable of evading protection conferred by traditional lineage I vaccines. However, due to the high genetic similarity between the vaccine strain and conventional genotype 1, the current assay cannot distinguish vaccine-induced antibodies from those produced during natural infection (i.e., it cannot differentiate between infected and vaccinated animals).

During the establishment and validation of this study, the positive clinical samples used were primarily derived from genotype 5 ARV. Future studies will systematically evaluate the detection performance across genotypes 1–4, 6, and 7, while also validating the applicability of this method in other poultry species (e.g., ducks and turkeys). In summary, the dual-antigen indirect ELISA established in this study effectively overcomes the limitations of traditional methods for detecting genotype 5 ARV. This method not only provides technical support for the targeted prevention and control of variant ARV strains but also establishes a theoretical foundation for the development of multiplex serological assays for other ARV genotypes.

## 5. Conclusions

In this study, a dual-antigen (σB and σC) indirect ELISA detection method was successfully established. In clinical application, it achieved a 100% detection rate for avian reovirus genotype 5-positive samples and enabled specific detection within 1 week post-infection, effectively monitoring dynamic antibody changes. The establishment of this method provides reliable technical support for the epidemiological surveillance and control of avian reovirus genotype 5.

## Figures and Tables

**Table 1:** Determination of optimal antigen coating concentration.

Antigen Used for Coating		Antigen Coating Concentration (μg/mL)					
		0.2	0.4	0.6	0.8	1	2
σB	P	0.476	0.556	0.800	1.204	1.495	1.926
	N	0.140	0.128	0.148	0.176	0.184	0.233
	P/N	3.406	4.330	5.396	6.843	8.140	8.256
σC	P	0.773	1.087	1.519	1.910	2.161	2.656
	N	0.137	0.141	0.151	0.172	0.171	0.233
	P/N	5.642	7.727	10.082	11.085	12.660	11.399
σB and σC	P	0.914	1.218	1.656	2.084	2.307	2.771
	N	0.139	0.139	0.148	0.169	0.191	0.236
	P/N	6.562	8.744	11.191	**12.333**	12.077	11.742

Notes. The black bold value indicates the value under the optimal condition chosen for subsequent indirect ELISA with dual antigen. P: OD value of positive samples; N: OD value of negative samples.

**Table 2:** Coating volume ratio of ET28a-σB and ET28a-σC.

Dilution of Serum		Molar Ratio of σB to σC (0.8 μg/mL)						
		1:1	1:2	1:3	1:4	2:1	3:1	4:1
1:50	P	1.954	2.149	2.490	2.308	1.910	1.869	1.805
	N	0.371	0.352	0.391	0.383	0.364	0.349	0.321
	P/N	5.268	6.100	6.369	6.022	5.242	5.361	5.624
1:100	P	1.686	1.774	2.128	2.009	1.695	1.600	1.508
	N	0.295	0.264	0.201	0.230	0.231	0.225	0.260
	P/N	5.708	6.712	10.587	8.721	7.338	7.110	5.791
1:200	P	1.552	1.605	1.815	1.772	1.509	1.486	1.450
	N	0.246	0.213	0.206	0.202	0.208	0.213	0.188
	P/N	6.316	7.552	8.825	8.509	7.265	6.997	7.715
1:400	P	1.415	1.525	1.739	1.644	1.386	1.306	1.243
	N	0.195	0.189	0.240	0.191	0.205	0.185	0.172
	P/N	7.242	8.083	8.997	8.594	7.246	7.702	7.215
1:600	P	1.319	1.483	1.689	1.568	1.280	1.267	1.209
	N	0.187	0.179	0.202	0.213	0.195	0.179	0.170
	P/N	7.038	8.300	**9.111**	8.415	7.043	7.352	7.098
1:800	P	1.219	1.370	1.550	1.431	1.248	1.195	1.123
	N	0.172	0.179	0.192	0.190	0.195	0.176	0.170
	P/N	7.087	7.668	8.501	7.821	7.013	7.045	6.754
1:1000	P	1.138	1.167	1.352	1.276	1.121	1.018	0.865
	N	0.178	0.177	0.184	0.179	0.192	0.173	0.171
	P/N	6.381	6.591	7.992	7.262	6.664	6.110	5.276
1:1200	P	0.941	1.087	1.202	1.164	0.925	0.875	0.785
	N	0.169	0.168	0.172	0.180	0.179	0.174	0.167
	P/N	5.568	6.455	7.196	6.730	5.581	5.206	4.824

Notes. The black bold value indicates the value under the optimal condition chosen for subsequent indirect ELISA with dual antigen. P: OD value of positive samples; N: OD value of negative samples.

**Table 3:** Results of the repeatability assay for the dual-antigen indirect ELISA.

Sample	No.	Inter-Assay		Intra-Assay	
		X ± SD	CV (%)	X ± SD	CV (%)
Positive serum samples	1	1.459 ± 0.070	4.8	0.789 ± 0.059	7.5
	2	1.326 ± 0.049	3.7	0.724 ± 0.056	7.7
	3	1.563 ± 0.086	5.5	0.906 ± 0.078	8.6
	4	0.986 ± 0.046	4.6	1.242 ± 0.084	6.8
	5	0.720 ± 0.024	3.3	0.642 ± 0.052	8.1
	6	1.706 ± 0.058	3.4	1.530 ± 0.112	7.3
Negative serum samples	7	0.200 ± 0.008	4.1	0.230 ± 0.016	7.1
	8	0.211 ± 0.006	2.9	0.237 ± 0.013	5.6
	9	0.186 ± 0.009	4.8	0.204 ± 0.012	6.0
	10	0.233 ± 0.014	5.9	0.275 ± 0.021	7.5
	11	0.117 ± 0.006	4.7	0.248 ± 0.018	7.1
	12	0.190 ± 0.008	4.1	0.242 ± 0.015	6.0

**Table 4:** Comparison of the dual-antigen ELISA and the commercial kit.

Dual-Antigen Indirect ELISA	Commercial Kit		Total
	Positive	Negative	
Positive	30	10	40
Negative	0	30	30
Total	30	40	70

## Data Availability

The original contributions presented in this study are included in the article.

## Supplementary Materials

The following supporting information can be downloaded at: https://www.mdpi.com/article/10.3390/ani16081273/s1, Figure S1. Phylogenetic relationships of the σC protein among genotype 5 ARV strains, including the LY383 and RG5 strains. The LY383 and RG5 strains are indicated with red dots. Multiple sequence alignment was performed using ClustalW, version 2.1, and the phylogenetic tree was constructed using the neighbor-joining method.

## Abbreviations

The following abbreviations are used in this manuscript:

AIV	Avian influenza virus
ALV	Avian leukosis virus
ARV	Avian reovirus
BSA	Bovine serum albumin
ELISA	Enzyme-linked immunosorbent assay
IBDV	Infectious bursal disease virus
IPTG	Isopropyl β-D-1-thiogalactopyranoside
NDV	Newcastle disease virus
