# Supplementary material for: Development of a Highly Sensitive ELISA for Detecting Antibodies Against a Novel Variant Avian Reovirus Based on Dual σC and σB Antigens
Source: Animals (Basel). 2026 Apr 21;16(8):1273. doi: 10.3390/ani16081273 (PMC13113393; doi:10.3390/ani16081273)
Supplement: Supplementary file 1 [file animals-16-01273-s001.zip › animals-4210905-supplementary.pdf]

Supplementary Material

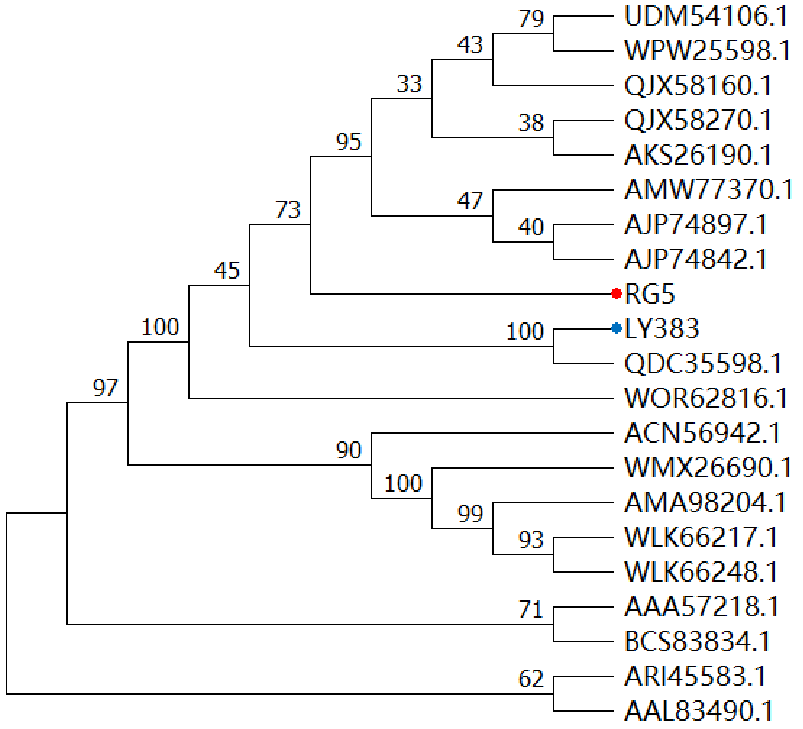

Figure S1. Phylogenetic analysis of the σC protein from genotype 5 avian reovirus strains. The LY383 and RG5 strains are marked with blue and red dots. Multiple sequence alignment was performed using ClustalW, and the phylogenetic tree was constructed using the neighbor-joining method.
